# Consumption of a High-Fat Diet in Adulthood Ameliorates the Effects of Neonatal Parathion Exposure on Acetylcholine Systems in Rat Brain Regions

**DOI:** 10.1289/ehp.0800459

**Published:** 2009-02-03

**Authors:** Theodore A. Slotkin, T. Leon Lassiter, Ian T. Ryde, Nicola Wrench, Edward D. Levin, Frederic J. Seidler

**Affiliations:** 1 Department of Pharmacology & Cancer Biology and; 2 Department of Psychiatry & Behavioral Sciences, Duke University Medical Center, Durham, North Carolina USA

**Keywords:** acetylcholine, brain development, high-fat diet, organophosphate insecticides, parathion

## Abstract

**Background:**

Developmental exposure to a wide variety of developmental neurotoxicants, including organophosphate pesticides, evokes late-emerging and persistent abnormalities in acetylcholine (ACh) systems. We are seeking interventions that can ameliorate or reverse the effects later in life.

**Objectives:**

We administered parathion to neonatal rats and then evaluated whether a high-fat diet begun in adulthood could reverse the effects on ACh systems.

**Methods:**

Neonatal rats received parathion on postnatal days 1–4 at 0.1 or 0.2 mg/kg/day, straddling the cholinesterase inhibition threshold. In adulthood, half the animals were switched to a high-fat diet for 8 weeks. We assessed three indices of ACh synaptic function: nicotinic ACh receptor binding, choline acetyltransferase activity, and hemicholinium-3 binding. Determinations were performed in brain regions comprising all the major ACh projections and cell bodies.

**Results:**

Neonatal parathion exposure evoked widespread abnormalities in ACh synaptic markers, encompassing effects in brain regions possessing ACh projections and ACh cell bodies. In general, males were affected more than females. Of 17 regional ACh marker abnormalities (10 male, 7 female), 15 were reversed by the high-fat diet.

**Conclusions:**

A high-fat diet reverses neurodevelopmental effects of neonatal parathion exposure on ACh systems. This points to the potential for nonpharmacologic interventions to offset the effects of developmental neurotoxicants. Further, cryptic neurodevelopmental deficits evoked by environmental exposures may thus engender a later preference for a high-fat diet to maintain normal ACh function, ultimately contributing to obesity.

Recent data indicate an alarming increase in the incidence of neurodevelopmental disorders [for review, see Grandjean and Landrigan 2006; Landrigan et al. 1994; Szpir 2006a, 2006b; Weiss et al. 2004; Weiss and Bellinger 2006)], currently involving up to 17% of U.S. school children, including attention deficit hyperactivity disorder, learning disabilities, and autism spectrum disorders, at an annual cost of $80–170 billion (Szpir 2006a, 2006b). Exposures to environmental chemicals are strongly suspected to play a key role in these increases, contributing to what has been termed a “silent pandemic” (Grandjean and Landrigan 2006). Given that thousands of new chemicals are introduced each year, most of which are untested for developmental neurotoxicity, it seems unlikely that exposures to such toxicants will decline. Accordingly, we already have a legacy of millions of children with environmentally related neuro-developmental disorders, and we can expect this problem to continue or even increase. Toxicologic research most often focuses on the identification of specific toxicants and their mechanisms of action, but it is equally valid to examine whether subsequent interventions can offset or ameliorate the consequences of exposure. In our previous work, we showed how identification of specific neurotransmitter or signaling pathways affected by diverse neurotoxicants can lead to therapies that restore both synaptic and behavioral function (Beer et al. 2005; Izrael et al. 2004; Shahak et al. 2003; Slotkin et al. 2001b; Steingart et al. 2000a, 2000b; Yanai et al. 2002, 2004, 2005); in particular, we were able to repair deficient function of acetylcholine (ACh) projections in the hippocampus by transplanting neural progenitor cells or to achieve pharmacologic reversal of symptoms by chronic treatment with ACh receptor agonists.

Here, we focus on a strategy that may be more readily applicable to neurodevelopmental disorders, namely dietary manipulation in which the majority of calories are derived from fat (i.e., a “ketogenic” diet). This approach has met with some success in drug-resistant childhood epilepsies, although the specific mechanism for the improvement is unknown (Bough and Rho 2007; Connolly et al. 2006; Hallbook et al. 2007; Hartman and Vining 2007). Interestingly, the same approach has been tried in treating attention deficit hyperactivity disorder and autism in pilot studies (Connolly et al. 2006; Evangeliou et al. 2003; Pulsifer et al. 2001), again with some evidence of success. However, it must be noted that such trials obviously cannot be blinded because the diet is altered in a way known to the subject. A few animal studies examined the effects of a high-fat diet on neural function, providing some evidence for a generalized decrease in excitability and reduced motor activity (Murphy and Burnham 2006; Murphy et al. 2005); notably, in keeping with the pilot human studies, high fat diets ameliorate behavioral anomalies in genetically modified mice (Teegarden et al. 2008). The ability of dietary manipulations to evoke widespread changes in neuro transmitter function is likely due to changes in the composition of membrane lipids in which receptors and cell signaling molecules are embedded (Gudbjarnason and Benediktsdottir 1996; Ponsard et al. 1999) and therefore can span multiple brain regions and neurotransmitter systems. Indeed, diet-induced changes in neural membrane lipids are known to alter neuro transmitter uptake and release, as well as function of neurotransmitter receptors and their signaling pathways (Clandinin et al. 1983; Geiser 1990; Kelly et al. 1995).

In the present study, we present a “proof of principle” by evaluating the ability of a high-fat diet to reverse the ACh-related synaptic abnormalities evoked by neonatal exposure to an organophosphate pesticide, parathion. Organophosphates are the most widely used insecticides (Casida and Quistad 2004) and human exposures are virtually ubiquitous (Casida and Quistad 2004; Morgan et al. 2005). These agents are undergoing increased scrutiny specifically because of their propensity to elicit developmental neurotoxicity at levels below those required for any signs of systemic exposure (Colborn 2006; Costa 2006; Pope 1999; Slotkin 2004, 2005). Here, we evaluated the effects of a brief neonatal parathion exposure at doses straddling the threshold for cholinesterase inhibition and the first signs of toxicity (Slotkin et al. 2006a). In adulthood, we switched some of the animals to a ketogenic, high-fat diet that more than doubles serum β-hydroxybutyrate concentrations (Lassiter et al. 2008). We performed assessments in brain regions encompassing major ACh projections as well as those containing the corresponding cell bodies, focusing on three markers of ACh synaptic function that are targeted by developmental exposure to parathion and that contribute to ACh-related behavioral impairment by organophosphates (Slotkin et al. 2006a, 2007a, 2008b; Timofeeva et al. 2008b): activity of choline acetyltransferase (ChAT), cell membrane binding of hemicholinium-3 (HC3) to the presynaptic high-affinity choline transporter, and the concentration of α4β2 nicotinic ACh receptors (nAChRs). ChAT is the enzyme that synthesizes ACh and, because it is a constitutive component of ACh nerve terminals, its activity provides an index of the development of ACh projections (Dam et al. 1999; Happe and Murrin 1992; Monnet-Tschudi et al. 2000; Qiao et al. 2003; Richardson and Chambers 2005; Slotkin et al. 2001a). Although HC3 binding to the choline transporter is also a constituent of ACh nerve terminals, its expression is directly responsive to neuronal activity (Klemm and Kuhar 1979; Simon et al. 1976), so that comparative effects on HC3 binding and ChAT enables the characterization of both the development of innervation and presynaptic activity. Last, the α4β2 nAChR is a key player in the ability of ACh systems to release other neuro transmitters involved in reward, cognition, and mood (Buisson and Bertrand 2001, 2002; Dani and De Biasi 2001; Fenster et al. 1999; Quick and Lester 2002) and is also the most abundant nAChR subtype in the mammalian brain (Flores et al. 1992; Happe et al. 1994; Whiting and Lindstrom 1987, 1988).

## Materials and Methods

### Animal treatments and diet

All experiments were carried out humanely and with regard for alleviation of suffering, with protocols approved by the Duke University Institutional Animal Care and Use Committee and in accordance with all federal and state guidelines. Timed-pregnant Sprague-Dawley rats (Charles River, Raleigh, NC) were housed in breeding cages, with a 12 hr light–dark cycle and free access to water and food (LabDiet 5001; PMI Nutrition, St. Louis, MO). On the day after birth, all pups were randomized and redistributed to the dams with a litter size of 10 (5 males, 5 females) to maintain a standard nutritional status. Parathion (99.2% purity; Chem Service, West Chester, PA) was dissolved in DMSO to provide consistent absorption (Slotkin et al. 2006a, 2006b; Whitney et al. 1995) and was injected subcutaneously in a volume of 1 mL/kg once daily on post-natal days 1–4; control animals received equivalent injections of the DMSO vehicle. Doses of 0.1 and 0.2 mg/kg/day were chosen because they straddle the threshold for barely detectable cholinesterase inhibition and the first signs of reduced weight gain or impaired viability (Slotkin et al. 2006a, 2006b). Brain cholinesterase inhibition 24 hr after the last dose of 0.1 mg/kg parathion is reduced 5–10%, well below the 70% threshold necessary for symptoms of cholinergic hyperstimulation (Clegg and van Gemert 1999). Randomization of pup litter assignments within treatment groups was repeated at intervals of several days up until weaning; in addition, dams were rotated among litters to distribute any maternal caretaking differences randomly across litters and treatment groups. Offspring were weaned on postnatal day 21.

Beginning at 15 weeks of age, half the rats were switched to a high-fat diet (OpenSource D12330; Research Diets Inc., New Brunswick, NJ), providing 58% of total calories as fat; 93% of the fat is hydrogenated coconut oil. The remaining rats continued on the standard LabDiet 5001 diet, which provides 13.5% of total calories as fat; with this diet, 27% of the fat is saturated. Although the high-fat diet contains 37% more calories per gram, we found that animals on this diet reduced their food intake by approximately the same proportion (Lassiter et al. 2008), so the total dietary intake is isocaloric; nevertheless, animals gained excess weight because of the higher fat content (Lassiter et al. 2008). During the 24th post natal week, animals were decapitated and brains were dissected into the frontal/parietal cortex, temporal/occipital cortex, hippo campus, striatum, midbrain, and brainstem. Neurochemical determinations were made on regions from six rats per treatment group for each sex and with each diet, with no more than one male and one female derived from a given litter in each group.

### Assays

Tissues were thawed in 79 volumes of ice-cold 10 mM sodium-potassium phosphate buffer (pH 7.4) and homogenized with a Polytron (Brinkmann Instruments, Westbury, NY). Duplicate aliquots of the homogenate were assayed for ChAT using established procedures (Qiao et al. 2003, 2004). Each tube contained 50 μM [^14^C]acetyl-coenzyme A (specific activity 6.7 mCi/mmol; PerkinElmer Life Sciences, Boston, MA) as a substrate, and activity was determined as the amount of labeled ACh produced relative to tissue protein (Smith et al. 1985).

For measurements of HC3 binding, the cell membrane fraction was prepared from an aliquot of the same tissue homogenate by sedimentation at 40,000 × *g* for 15 min. The pellet was resuspended and washed, and the resultant pellet was assayed by established procedures (Qiao et al. 2003, 2004), using a ligand concentration of 2 nM [^3^H]HC3 (specific activity, 125 Ci/mmol; PerkinElmer) with or without 10 μM unlabeled HC3 (Sigma Chemical Co., St. Louis, MO) to displace specific binding. Determinations of nAChR binding were carried out in another aliquot, each assay containing 1 nM [^3^H]cytisine (specific activity 35 Ci/mmol; PerkinElmer) with or without 10 μM nicotine (Sigma) to displace specific binding (Slotkin et al. 2008a). Binding was calculated relative to the membrane protein concentration.

### Data analysis

Data were compiled as means ± SE. Because we evaluated three neuro chemical measures that were all related to ACh synapses, the initial comparisons were conducted by a global analysis of variance (ANOVA; data log-transformed because of heterogeneous variance among regions and measures) incorporating all the variables and measurements in order to avoid an increased probability of type 1 errors that might otherwise result from multiple tests of the same data set. The variables in the global test were treatment (control, parathion 0.1 mg/kg, parathion 0.2 mg/kg), diet (normal, high-fat), brain region, sex, and measure (nAChR binding, ChAT, HC3 binding); the latter was considered a repeated measure because all three determinations were derived from the same sample. Where we identified interactions of treatment with the other variables, data were then subdivided for lower-order ANOVAs to evaluate treatments that differed from the corresponding control. Where permitted by the interaction terms, individual groups that differed from control were identified with Fisher’s protected least significant difference test. Significance was assumed at the level of *p*< 0.05.

To ensure that treatment and diet effects could be compared across all groups, we conducted all three assays simultaneously on all samples for a given region and sex, but technical limitations dictated that each region and sex had to be performed in divided runs. Accordingly, the control values for region versus region or for males versus females cannot be compared directly, since each region was assayed separately, as was each sex. However, treatment and diet effects and their interactions with region and sex can be interpreted because these depend solely on the internal comparison to the matched control groups that were run together. In evaluating the magnitude of the changes elicited by parathion administration, we used entire brain regions rather than specific nuclei, which means that even drastic effects on a specific population of neurons show up as smaller changes due to dilution with unaffected areas. Despite this limitation, we found statistically significant alterations for both treatment paradigms in multiple regions.

## Results

The global ANOVA identified a main effect of parathion treatment (*p* < 0.0001) as well as interactions of treatment with all the other variables: *p* < 0.01 for treatment × sex, *p* < 0.0001 for treatment × ACh measure, *p* < 0.007 for treatment × sex × diet, *p* < 0.05 for treatment × diet × region, *p* < 0.002 for treatment × sex × ACh measure, *p* < 0.05 for treatment × region × ACh measure, *p* < 0.0001 for treatment × sex × diet × ACh measure, *p* < 0.006 for treatment × diet × region × ACh measure, and *p* < 0.05 for treatment × sex × diet × region × ACh measure. In light of these interactions and of previous findings of strong sex differences in the effects of neonatal parathion exposure on neurodevelopment (Lassiter et al. 2008; Slotkin et al. 2008b, 2009), we also conducted a lower-order test separately for males and females. Again, parathion treatment interacted with each of the other variables, but on the whole, the effects in males were more statistically robust than those in females (males: *p* < 0.0001 for the main effect of treatment, *p* < 0.0001 for treatment × ACh measure, *p* < 0.02 for treatment × diet, *p* < 0.03 for treatment × region, *p* < 0.01 for treatment × diet × ACh measure, *p* < 0.002 for treatment × diet × region × ACh measure; females: *p* < 0.0002 for treatment × diet × ACh measure, *p* < 0.002 for treatment × diet × region × ACh measure). As required by these inter actions, we then separated the results according to region, diet, sex, and ACh measure for presentation.

For the control group, the high-fat diet alone had no statistically significant effect on any of the measures when evaluated in a global test (factors of diet, sex, region, ACh measure) or separately for each of the measures. Accordingly, where apparent changes are caused by diet alone in the control group, the overall incidence of such “differences” cannot be distinguished from random; indeed, in the global ANOVA, we found no main effect of diet or interaction of diet × region (exclusive of the interactions with parathion treatment) for males or females for any of the variables. Further, because binding affinity can be influenced by the lipid milieu in which the proteins are embedded (Gudbjarnason and Benediktsdottir 1996; Ponsard et al. 1999), the effects of parathion must be compared to the corresponding, diet-matched control group, whereas interpreting apparent differences in the two control groups is problematic.

**Figure 1:**
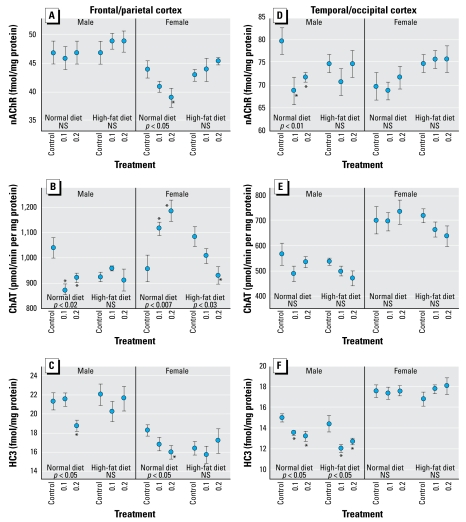
Effects of neonatal parathion exposure (0.1 or 0.2 mg/kg) and subsequent adult consumption of a high-fat diet on ACh synaptic parameters in the frontal/parietal cortex (*A–C*) and temporal/occipital cortex (*D–F*). (*A, D*) nAChR binding. (*B, E*) ChAT activity. (*C, F*) HC3 binding to the presynaptic high-affinity choline transporter. Data represent mean ± SE obtained from six rats per group. ANOVA for each cluster appears within the panels. NS, not significant. *Individual values for which the parathion groups differ from the corresponding diet-matched control.

Parathion had differential effects on the ACh measures in the various brain regions of males and females, and most of these were reversed by the high fat diet. In the frontal/parietal cortex, neonatal parathion exposure had little or no effect on nAChR binding in males, regardless of whether animals were consuming a normal or high-fat diet (Figure 1A). In females, parathion evoked a dose-dependent decrement in frontal/parietal cortical nAChR binding that was completely reversed by a high-fat diet. For ChAT activity in frontal/parietal cortex, males showed significant reductions at either parathion dose; again, the high-fat diet reversed the defects (Figure 1B). In females, neo natal parathion exposure elicited substantial increases in ChAT in the same region; in this case, the high-fat diet completely reversed the pattern, such that animals exposed to 0.2 mg/kg parathion exhibited significantly lower values than controls. For HC3 binding in the frontal/parietal cortex, neonatal parathion exposure caused significant reductions in both males and females, whereas animals on a high-fat diet did not display any deficits (Figure 1C). In the temporal/occipital cortex, neonatal parathion exposure elicited significant reductions in nAChR binding in males on the normal diet but not in those on the high-fat diet; we saw no parathion effects in females on either diet (Figure 1D). Also in this region we observed no significant changes in ChAT with parathion alone or in combination with a high-fat diet (Figure 1E). For HC3 binding in the temporal/occipital cortex, males showed a significant reduction caused by neonatal parathion; however, for this parameter, the high-fat diet provided no protection (Figure 1F); females showed no significant effects on HC3 binding.

**Figure 2:**
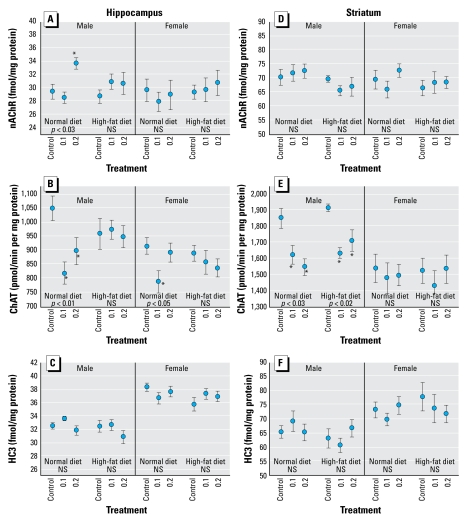
Effects of neonatal parathion exposure (0.1 or 0.2 mg/kg) and subsequent adult consumption of a high-fat diet on ACh synaptic parameters in the hippocampus (*A–C*) and striatum (*D–F*). (*A, D*) nAChR binding. (*B, E*) ChAT activity. (*C, F*) HC3 binding to the presynaptic high-affinity choline transporter. Data represent mean ± SE obtained from six rats per group. ANOVA for each cluster appears within the panels. NS, not significant. *Individual values for which the parathion groups differ from the corresponding diet-matched control.

In the hippocampus, males exposed to the high dose of parathion displayed significant elevations in nAChR binding that were reversed by the high-fat diet (Figure 2A); females showed no significant effects of parathion in either dietary group. In animals consuming a normal diet, hippocampal ChAT was significantly reduced at both doses in males and at the low dose in females, but no such changes were seen on the high-fat diet (Figure 2B). In contrast, hippocampal HC3 binding was unaffected by parathion with or without a high-fat diet (Figure 2C). In the striatum, nAChR binding was unaffected by parathion exposure (Figure 2D). However, for striatal ChAT, parathion evoked significant reductions in males but not females, and the high-fat diet was unable to reverse the effect (Figure 2E). Striatal HC3 binding evinced no significant differences (Figure 2F).

**Figure 3:**
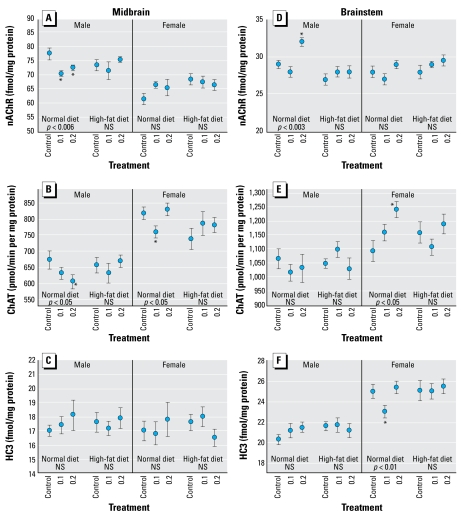
Effects of neonatal parathion exposure (0.1 or 0.2 mg/kg) and subsequent adult consumption of a high-fat diet on ACh synaptic parameters in the midbrain (*A–C*) and brainstem (*D–F*). (*A, D*) nAChR binding. (*B, E*) ChAT activity. (*C, F*) HC3 binding to the presynaptic high-affinity choline transporter. Data represent mean ± SE obtained from six rats per group. ANOVA for each cluster appears within the panels. NS, not significant. *Individual values for which the parathion groups differ from the corresponding diet-matched control.

In the midbrain, males on the normal diet showed a significant parathion-induced reduction in nAChR binding that was not seen in animals on the high-fat diet (Figure 3A); females showed no significant effects. For midbrain ChAT, both sexes showed significant reductions evoked by neonatal parathion exposure, involving the high dose for males and the low dose for females (Figure 3B); the high-fat diet eliminated both defects. Midbrain HC3 binding was generally unaffected by parathion (Figure 3C). In the brainstem, neonatal parathion exposure evoked nAChR up-regulation restricted to males, and once again, the high-fat diet completely reversed the effect (Figure 3D). In contrast, parathion-exposed females but not males showed elevated ChAT (Figure 3E) and suppressed HC3 binding (Figure 3F) in the brainstem, and both of these effects were offset by the high-fat diet.

We presented the effects of parathion treatments and dietary manipulations on body weights in this model previously (Lassiter et al. 2008); because we used animals from the same cohort in the present study, the results are not presented here. In brief, parathion alone produced a small (2–3%) but significant elevation in weight at the low dose in males and reductions of about 4% at either dose in females. The high-fat diet alone produced significant increases in body weight for both males (10% increase) and females (30% increase). In males, neonatal parathion treatment did not affect the body weight response to high fat, whereas the dietary effect was diminished at the high parathion dose in females.

## Discussion

Our results clearly indicate that consuming a high-fat diet in adulthood can ameliorate many of the long-term ACh synaptic abnormalities evoked by neonatal parathion exposure. We assessed three ACh measures for each of six brain regions, for a total of 18 sets of determinations for each sex. In males, parathion exposure evoked significant changes in 10 of these measures, of which 8 were reversed by the high-fat diet; in females, 7 of the measures were affected, and all of them were reversed by dietary manipulation, albeit that one measure then became abnormal in the opposite direction (ChAT in the frontal/parietal cortex). These findings provide a proof of principle that dietary interventions are capable of offsetting the ACh synaptic defects caused by developmental neurotoxicant exposure; future studies clearly need to address whether behavioral performance is similarly restored by dietary manipulation, as is the case for anomalies evoked by genetic manipulations (Teegarden et al. 2008).

Our results thus open a new avenue for developing general amelioration strategies that may prove useful for diffuse and diverse neurotoxicants. Obviously, the use of a high-fat diet poses serious metabolic problems that may preclude its generalized use. Indeed, in our earlier work, we showed that neonatal exposure to organophosphates evokes long-term changes in metabolic function that contribute to obesity, prediabetes, and cardiovascular risk factors such as elevated serum lipids (Lassiter and Brimijoin 2008; Lassiter et al. 2008; Roegge et al. 2008; Slotkin et al. 2005). The metabolic abnormalities were exacerbated by a high-fat diet (Lassiter et al. 2008; Roegge et al. 2008). It will therefore be important to establish whether there are specific components of the diet that are the key elements responsible for the reversal of ACh synaptic abnormalities that may permit use of a less injurious dietary manipulation.

Nevertheless, the effects of parathion on ACh systems comprise an alteration in the “trajectory” of ACh synaptic development and function, rather than representing an outright initial injury that simply continues into adulthood. Indeed, none of these synaptic changes is apparent in the immediate posttreatment period (Slotkin et al. 2006a); instead, the effects emerge over an extended time frame ranging from adolescence to adulthood (Slotkin et al. 2008b, 2009). This means that there may be specific developmental “windows” in which a short-term intervention can redirect the trajectory of ACh synaptic development, thus avoiding the need for life-long intervention. Again, this will be an important subject for future studies.

The results of the present study also confirm and extend earlier work on the developmental neuro toxicity of parathion that indicated sex-selectivity and regional differences in the effects on ACh systems as well as other neuro transmitters (Slotkin et al. 2008b, 2009). Developmental exposure to organophosphates commonly elicits highly sex-dependent alterations at the synaptic and behavioral levels (Aldridge et al. 2004, 2005; Dam et al. 2000; Levin et al. 2001, 2002; Moser et al. 1998; Ricceri et al. 2006; Roegge et al. 2008; Slotkin et al. 2001a, 2002a, 2006b, 2008a, 2008c; Slotkin and Seidler 2005, 2007; Timofeeva et al. 2008a) because of interference with sexual differentiation of the brain (Aldridge et al. 2005; Levin et al. 2001; Slotkin 1999, 2004, 2005). However, in comparing our results in the present study at 5 months of age to those seen in adolescence (1 month of age) or young adulthood (2–3 months of age) (Slotkin et al. 2008b), we observed that many of the sex-selective differences intensified with time. This is consonant with the fact that, even where an initial injury might be equivalent in males and females, subsequent repair processes are generally greater in females, thus contributing to even further differences in the trajectory of ACh synaptic development and function (Amateau and McCarthy 2002; Hilton et al. 2004; McEwen 2002; Nunez and McCarthy 2003; Slotkin et al. 2007b; Tanapat et al. 1999). Similar effects are likely to play a role in the fact that the high-fat diet successfully reversed all of the parathion-induced changes in females, whereas some of the abnormalities persisted in males.

As in a number of earlier studies of the effects of developmental organophosphate exposure (Levin et al. 2002; Slotkin et al. 2008a; Timofeeva et al. 2008a), we found some effects of parathion that were nonmonotonic, with significant alterations at the low dose but not at the high dose (ChAT in female hippocampus and midbrain, HC3 binding in female brainstem). This likely represents the fact that the higher dose of parathion elicits some systemic toxicity (Slotkin et al. 2006a), which will by itself produce additional changes in ACh function. Additionally, cholinesterase inhibition at the higher dose can provide a positive trophic effect by increasing the levels of ACh in the developing brain (Hohmann 2003; Lauder and Schambra 1999; Picciotto and Zoli 2008; Slotkin et al. 2006b). Indeed, a carefully chosen, small dose of chlorpyrifos can actually enhance some aspects of neurodevelopment while damaging other components (Laviola et al. 2006). In the present study, the high-fat diet reversed the anomalies regardless of whether they were nonmonotonic or monotonic, so the presence or absence of these additional components does not appear to be critical to the ameliorating effect of dietary manipulation.

Finally, results of the present study point to an important consideration in the explosive worldwide increase in obesity. It is clear that neurodevelopmental disorders can influence apparent lifestyle choices, most notably in the increased incidence of drug abuse or cigarette smoking (Deas and Brown 2006; Kandel et al. 1994; Pliszka and Pliszka 2003; Weissman et al. 1999; Wilens 2004). In a recent study Jacobsen et al. (2007) showed that abstinence from smoking in adolescent smokers whose mothers smoked during pregnancy leads to cognitive impairment, whereas those who were born to nonsmokers showed cognitive improvement upon abstinence from smoking. In other words, where there was preexisting neurodevelopmental damage from prenatal tobacco exposure, adolescents were able to offset cognitive impairment by smoking; this likely contributes to the higher likelihood of the children born to smoking women becoming smokers themselves (Kandel et al. 1994; Weissman et al. 1999). By the same token, our studies point to the possibility that exposure to developmental neuro toxicants could contribute to a subsequent preference for a high-fat diet as a way of ameliorating the effects, thus providing an indirect but potentially potent driving force for consuming an unhealthy diet. If this turns out to be true, then our findings point to a potentially important contributory factor in the increased incidence of obesity and diabetes, expanding the public health implications of the “silent pandemic” caused by developmental neurotoxicant exposure (Grandjean and Landrigan 2006).
